# Intrahospital transport of critically ill patients (excluding newborns) recommendations of the Société de Réanimation de Langue Française (SRLF), the Société Française d'Anesthésie et de Réanimation (SFAR), and the Société Française de Médecine d'Urgence (SFMU)

**DOI:** 10.1186/2110-5820-2-1

**Published:** 2012-02-03

**Authors:** Jean-Pierre Quenot, Christophe Milési, Aurély Cravoisy, Gilles Capellier, Olivier Mimoz, Olivier Fourcade, Pierre-Yves Gueugniaud

**Affiliations:** 1 Service de Réanimation Médicale, CHU Bocage Central Gabriel, 14 rue Paul Gaffarel, 21 079 Dijon, France; 2 Service de Réanimation Pédiatrique, CHU Lapeyronie, 371 avenue du doyen Gaston Giraud, 34 295 Montpelier, France; 3 Service de Réanimation Médicale, CHU Hôpital Central, 29, avenue du Maréchal de Lattre de Tassigny, 54 035 Nancy, France; 4 Service de Réanimation Médicale, CHU Hôpital Jean Minjoz, 3, Boulevard Fleming, 25 000 Besançon, France; 5 Service d'Anesthésie Réanimation, CHU de la Milétrie, 2 rue de la Milétrie, 86 021 Poitiers, France; 6 Pôle Anesthesie Réanimation, CHU pavillon urgences et réanimation, Hôpital Purpan, place du Docteur Baylac, 31 059 Toulouse, France; 7 Service Aide Médicale Urgente, CHU hospices civils, 162, avenue Lacassagne, 69 003 Lyon, France

**Keywords:** intrahospital transport, critical care, adults

## Abstract

Critically ill adult patients often require multiple examinations in the hospital and need transport from one department to another, or even between hospitals. However, to date, no guidelines exist regarding optimum practices for transport of these fragile patients. We present recommendations for intrahospital transport of critically ill patients, excluding newborns, developed by an expert group of the French-Language Society of Intensive Care (Société de Réanimation de Langue Française (SRLF), the Société Française d’Anesthésie et de Réanimation (SFAR), and the Société Française de Médecine d’Urgence (SFMU). The recommendations cover five fields of application: epidemiology of adverse events; equipment, monitoring, and maintenance; preparation of patient before transport; human resources and training for caregivers involved in transport processes; and guidelines for planning, structure, and traceability of transport processes.

## Introduction and methodology of the expert recommendations

These recommendations were developed by a Working Group brought together at the initiative of the French-Language Society of Intensive Care (Société de Réanimation de Langue Française (SRLF)). The experts comprising this group wrote a background text justifying each of the five fields of application that were previously defined by the Organizing Committee. The recommendations for pediatric patients were included in the relevant fields alongside the recommendations for adults. These expert recommendations constitute a contribution to the standard risk evaluation protocol and to the quality of care improvement program elaborated by professional societies in our discipline. The recommendations are mainly based on data from prospective or retrospective observational studies and international consensus documents. The recommendations are proposed and discussed individually, with each expert (or expert subgroup) obliged to provide scientific evidence to justify the basis for the recommendation, as well as the level of recommendation, each of which was subject to modification according to the remarks made by the rest of the expert group. In a second phase, the recommendations were graded by the whole expert group. The objective was not necessarily to arrive at a single consensus on the level and grade of recommendation for all the guidelines but rather to clearly identify areas where opinions converged, which would be the basis for the recommendations, as well as areas where disagreement persisted, which could be the object of further research.

Each recommendation was graded by each expert according to the RAND/UCLA appropriateness rating method, using repeated rounds of grading after exclusion of the extreme values (highly deviant expert ratings). Each expert graded recommendations based on a scale from 1 to 9 (with 1 corresponding to the existence of "total disagreement, " "absence of proof, " or "formal contraindication" and 9 corresponding to "total agreement, " "formal proof, " or "formal indication").

Three zones were defined according to the location of the median score: 1-3 corresponds to the disagreement zone; 4-6 to the zone of indecision; and 7-9 to the agreement zone. The level of agreement, indecision, or disagreement is considered to be "strong" if the median falls within the boundaries of the corresponding zone. If the medial overlaps with a boundary (e.g., interval from 1-4 or 6-8), then the agreement (or disagreement) is considered to be weak. The methodology for this document is based on the GRADE method http://www.gradeworkinggroup.org//links.htm.

The originality of the GRADE system resides in the following elements: the type of study design (i.e., randomized, controlled trials, or not) alone is not sufficient to attribute a level of evidence; the actual risk-benefit ratio is taken into account, and finally, recommendations are formulated clearly and unambiguously for users (we recommend/we do not recommend; we suggest/we do not suggest).

## Field 1

### Epidemiology of adverse events related to patients and their environment: general taxonomy

1) Critically ill patients, whether or not they are hospitalized in critical care, frequently require intrahospital transport (IHT) for diagnostic or therapeutic procedures, or for admission to a specialized care unit. Strong Agreement.

2) Critically ill patients include all patients presenting with dysfunction or failure or one or more vital organs or systems. Strong Agreement.

3) It is necessary to standardize the definitions of adverse events (AE) and their avoidance during IHT. Strong Agreement.

4) Adverse events are classified into two categories, according to their seriousness: serious adverse events (SAEs), and high-risk events (HREs). Strong Agreement.

5) An SAE is a complication directly related to patient care that can lead to a life-threatening situation, longer hospital stay, need for invasive procedures, or have serious consequences. Strong Agreement.

6) High-risk events are defined by current legislation for the accreditation of physicians practicing in at-risk disciplines. They cover all adverse events that are not SAE, i.e., minor adverse events and dysfunctions in equipment or organization of care. Strong Agreement.

7) Minor incidents without major consequences regularly occur during transport. Therefore, it is necessary to define the most common SAE and HRE to implement monitoring and corrective measures. Strong Agreement.

8) Adverse events that occur during transport and require curative therapeutic intervention must be considered SAEs. Strong Agreement.

9) Adverse events that occur during transport and require therapeutic intervention that does not succeed in correcting the situation according to the objectives laid down by the clinician must be considered as SAEs. Strong Agreement.

10) A HRE complicated by auto-extubation and/or cardiac arrest is a SAE. Strong Agreement.

11) Oxygen desaturation that requires an increase of fractional inspired oxygen (FIO_2_) or any other change in ventilator settings is considered a HRE. This should be considered a SAE if the situation remains uncorrected or does not reach the target set by the clinician. Strong Agreement.

12) A decrease in blood pressure that requires therapeutic intervention is a HRE. This should be considered a SAE if treatment does not reach the target set by the clinician. Strong Agreement.

13) A state of agitation or when the patient is not synchronized to the ventilator, a state that requires therapeutic intervention is considered a HRE. This should be considered a SAE if the situation remains uncorrected by the therapeutic intervention. Strong Agreement.

14) Any ventilator-related event that requires a change in ventilatory settings, ventilation with manual resuscitator (bag valve mask), or a change of equipment is considered a HRE. Strong Agreement.

15) Any ventilator-related event complicated by a clinical event is considered a SAE. Strong Agreement.

16) Disconnection of equipment (catheters, drains, intracranial pressure wires...) are considered HREs when they have no direct clinical consequences. They must be considered SAEs if a complication occurs. Strong Agreement.

17) An adverse event that occurs during IHT must be considered unavoidable when the entire transport process was performed in conformity with standard protocol. Strong Agreement.

18) Safety practices are any healthcare, structural, or organizational practices that contribute to preventing, reducing the frequency of, or attenuating the consequences of errors and adverse events during IHT. Strong Agreement.

19) Any adverse event that occurs during IHT should be notified for subsequent analysis. Strong Agreement.

## Field 2

### Equipment, monitoring and maintenance

1) The choice of equipment should take into account its bulk and autonomy. Strong Agreement.

2) All connections between the various monitors (e.g., invasive pressure wires) should be checked thoroughly before IHT. Strong Agreement.

3) The minimum monitoring required during IHT includes ECG heart rate monitoring, pulse oximetry, and noninvasive blood pressure monitoring. Strong Agreement.

4) In nonventilated patients, ventilatory rate should be monitored at regular intervals, ideally with continuous monitoring. Strong Agreement.

5) End-tidal CO_2 _(ET CO_2_) monitoring is recommended for patients with neurological disorders and for patients in whom strict control of partial pressure of CO_2 _(PaCO_2_) is required. Strong Agreement.

6) The main parameters being monitored should be associated with alarms whose settings can be adapted in each patient. Strong Agreement.

7) Special equipment should be available, dedicated to IHT, and clearly identified within each healthcare establishment, department, or division. Strong Agreement.

8) Ventilators used for transport should be equipped with visual or audible alarms for the main ventilatory parameters being monitored. Strong Agreement.

9) For ventilated patients undergoing transport that could be of long duration, or in patients at particularly high risk, a suction system should be immediately available, ideally in the form of a portable electric suction device. Strong Agreement.

10) The autonomy of all devices, in terms of electricity and medical gas supply, should be adapted to the estimated duration of IHT and rate of consumption, which can vary depending on usage, and reserves should be monitored. Strong Agreement.

11) Monitoring equipment should be adapted to the type of transport, patient risk, and ongoing therapy, and based on a written protocol. Strong Agreement.

12) Manual ventilation with a manual resuscitator (bag valve mask) during IHT should be avoided and only used in case of failure of the ventilator (including in children). Strong Agreement.

13) The settings on portable ventilators for use during transport should allow for the same ventilatory parameters as the ICU ventilator, including noninvasive ventilation modes. Strong Agreement.

14) At all times during transport, it should be possible, in ventilated patients, to switch immediately from ventilation to manual ventilation through an endotracheal tube or mask. Strong Agreement.

15) The exact capacities of the portable ventilator for use during transport should be known to the user. There are three categories. Strong Agreement.

- Basic or emergency ventilators (volume-control (VC) mode, positive end-expiratory pressure (PEEP), reduced monitoring)

- Intermediate ventilator (volume assist control (VAC), PEEP, adjustable flow or I:E ratio, spirometry), FiO_2 _setting at 100% or air/oxygen mix

- High-performance ventilator (volumetric and barometric ventilation modes, including spontaneous mode and assist control, PEEP, wide range of settings for FiO_2_, adjustable inspiratory flow, appropriate triggers, spirometry, ideally with circuit compliance compensation and non-invasive ventilation (NIV) mode).

16) The functions, monitoring, and alarms on the ventilator should be adapted to the patient's condition. Strong Agreement.

- A. Very hypoxemic patient (e.g., acute respiratory distress syndrome): high-performance ventilator

- B. Patient requiring strict control of PaCO_2_: intermediate or high-performance ventilator

- C. Patient-triggered ventilation (assist modes): intermediate or high-performance ventilator

- D. Patients under noninvasive ventilation: Ventilator with a high-performance NIV mode

17) The type of electric supply and recharging capabilities of the ventilator must be compatible with use at all times and should have sufficient electricity reserves to perform the planned IHT. Strong Agreement.

18) The ventilator used for IHT must have an audible alarm to signal interruption of gas or electricity supply, or ventilator failure. Strong Agreement.

19) The interface of the portable ventilator used for transport should not allow for any accidental disturbances to the ventilator settings. Strong Agreement.

20) At equal performance levels. Strong Agreement

- The ventilator with the simplest user interface should be given precedence.

- The ventilator with the simplest patient circuit should be given precedence.

21) To check that tolerance of the portable ventilator and patient stability are adequate, the portable ventilator should be connected to the patient 5 to 10 minutes before leaving the patient's room, using the wall gas supply and the mains electricity supply. Strong Agreement.

22) The portable ventilator should be stored in an easily accessible place that is known to all potential users and with all accessories: complete patient circuit kit with heat and moisture exchanger (HME) and corrugated tube, gas supply tube. Strong Agreement.

23) The circuit used should be in accordance with manufacturer's recommendations. Strong Agreement.

24) Where necessary, the ventilator tubes used should be adapted to the characteristics of the ventilator. Strong Agreement.

25) To ensure adequate humidification of the patient's airways and protection of the ventilator, an antibacterial filter and HME should be systematically put in place between the corrugated tube and the patient circuit. Strong Agreement.

26) The machine settings and alarms for the portable ventilator must be specified on a written prescription. Strong Agreement.

27) Ventilation monitoring by the portable ventilator should comprise, as a minimum requirement, monitoring of inspiratory pressure with display of the peak pressure and spirometry. Strong Agreement.

28) Self- or accidental extubation must be detected immediately by monitoring of capnography and/or spirometry. Strong Agreement.

29) Analysis of the expiratory phase of the capnogram can help to identify certain complications of ventilation during transport. Strong Agreement.

30) In synchronous intermittent mandatory ventilation (SIMV) mode, the portable ventilator should be equipped with the necessary general requirements in terms of performance and monitoring to guarantee appropriate ventilation. Strong Agreement.

31) Certain models of portable ventilator claim to be equipped with SIMV mode but in actual fact do not really provide this mode of ventilation. These ventilators should not be used. Strong Agreement.

32) Continuous positive airway pressure (CPAP) mode is suboptimal on ventilators and consumes large amounts of oxygen. Strong Agreement.

33) An invasive device for continuous measurement of blood pressure must be used during IHT if the patient is under treatment with vasoactive agents and/or hemodynamically unstable, and if the patient already has continuous invasive blood pressure monitoring in the hospital before IHT. Strong Agreement.

34) Monitoring of central venous pressure is not recommended during IHT. Strong Agreement.

35) A defibrillator-pacemaker must be easily available during transport. Ideally, it should be integrated with a multiparameter monitor. Strong Agreement.

36) If the patient is dependent on an external pacemaker, the thresholds of the pacemaker must be verified and adapted, and the battery should be checked. A spare external pacemaker must be available during transport. Strong Agreement.

37) In the presence of temporary pacing wires, a portable pacemaker must be used. Strong Agreement.

38) A written protocol must be put in place to plan for the immediate replacement of any defective or missing equipment. Strong Agreement.

39) The equipment used for IHT must be controlled regularly against to a predefined checklist. Strong Agreement.

40) After use, the ventilator must be cleaned and disinfected according to a written protocol. Strong Agreement.

41) During IHT of pediatric patients, a complete kit comprising resuscitation equipment and drugs for children must accompany the patient, particularly a self-inflating bag, a face mask, and an intubation kit adapted to the age of the child, as well as an intraosseous catheterization kit. Strong Agreement.

42) Monitoring of EtCO_2 _is recommended during transport in case of manual ventilation of an intubated child to prevent hyperventilation. Strong Agreement.

43) For the transport of children < 15 kg, it is mandatory to have a ventilator that can deliver low tidal volumes, ensure high frequencies, and maintain PEEP. Strong Agreement.

44) The size and compliance of the tubes used should be adapted to the age and weight of the child to minimize the compressible volume (small tubes for bodyweight < 15 kg). Strong Agreement.

## Field 3

### Preparation of the patient before transport

1) Before IHT, evaluation of the clinical status of the patient and the risk-benefit ratio must be performed. The results of these evaluations must be recorded in the patient's medical file. Strong Agreement.

2) It is mandatory to check for the absence of contraindications to complementary procedures. Strong Agreement.

3) The patient must be wearing an identification wristband. Strong Agreement.

4) At least one permeable venous access is required, and if necessary, an additional access line specifically reserved for amines and clearly identified as such. All access lines (central or peripheral) should be clean and firmly attached. Strong Agreement.

5) Electric syringe pumps should be clearly identified and the quantity of drugs adapted to the duration of transport. The electricity supply cables should be available during transport. Electric syringe pumps should be plugged back into a mains supply as soon as possible (as should all other electric material). Strong Agreement.

6) For patients who require strict control of PaCO_2_, an arterial sample should be taken before IHT to measure PaCO_2 _gradient and EtCO_2_. Strong Agreement

7) Cerebral perfusion pressure should continue to be monitored during transport of neurology patients. Strong Agreement.

8) The optimal patient position in critical care should be maintained during IHT. Strong Agreement.

9) Any pain that could be induced by IHT and/or the procedures to be performed should be anticipated, evaluated, and treated. Strong Agreement.

10) Sedation and/or analgesia should be maintained during transport and can be modified if necessary. Strong Agreement.

11) Mobilization of curarized patients should be the object of particular caution. Strong Agreement.

12) A complete intubation kit (including Eschmann tracheal tube introducer) should be immediately available. Strong Agreement.

13) Each time the patient is mobilized, a thorough verification of the correct positioning of all invasive devices should be performed. Strong Agreement.

14) A manual resuscitator (bag valve mask) with appropriate mask, oxygen reservoir, oxygen extension tube, and spare filter must accompany the patient during IHT. Strong Agreement.

15) Hypothermia during IHT must be avoided, particularly in children, by monitoring temperature. Strong Agreement.

16) Disposable equipment should be preferred. Strong Agreement.

17) The balloon pressure of the intratracheal tube should be verified before IHT and after IHT. Strong Agreement.

18) An emergency intervention kit should accompany the patient during transport. Strong Agreement.

## Field 4

### Caregivers: human resources and training

1) Initial and regular training is mandatory for all staff providing IHT, both in the use of equipment and its monitoring (ventilator, multiparametric monitors, defibrillator...). Strong Agreement.

2) Special initial and regular training is required for all medical and paramedical staff performing transport of pediatric patients < 15 kg. Strong Agreement.

3) Training of staff responsible for IHT can be provided in the form of simulations of transport situations. Strong Agreement.

4) Evaluation of risk and the specific modalities of IHT are the responsibility of the senior physician in charge of the patient. Strong Agreement.

5) The transport team for a critically ill patient must include at least one experienced physician and one staff member specially trained in IHT procedures. Strong Agreement.

6) A procedure for activating emergency aid (medical or nursing backup) should be available and known to all staff members, in case of any problem occurring during IHT. Strong Agreement.

7) An infusion specialized, or qualified equivalent, must be included in the IHT team when the patient is under extracorporeal circulation. Strong Agreement.

8) If the mobile medical emergency unit is responsible for performing IHT, then they should receive a full briefing on the status of the patient from the senior physician in charge of the patient, as well as a written report. Strong Agreement.

## Field 5

### Organization (planning), structure, and traceability

1) The exact time, meeting point, and duration of immobilization of the patient must be specified and checked before initiating IHT. Strong Agreement.

2) The name of the physician and the technical facilities available at the destination must be known in advance. Strong Agreement.

3) Before initiating IHT, the planned route must be mapped out and known to the transport staff, with knowledge of the accessibility of corridors and elevators, giving precedence to the shortest and safest route possible. Strong Agreement.

4) The destination department must be informed of the patient's imminent arrival. Strong Agreement.

5) The traceability of the variables monitored during IHT must be specifically recorded on a monitoring sheet that must subsequently be integrated into the patient's medical file. Strong Agreement.

6) A paper printout of traceability data (data monitored during IHT) is preferable. Strong Agreement.

7) If the physician that receives the patient at the destination is qualified to monitor the patient, the IHT team should transfer all necessary information to this physician to ensure continued care. Strong Agreement.

8) In the absence of a physician qualified to monitor the patient at the destination, the IHT team will remain in charge of monitoring the patient. Strong Agreement.

9) The organization of IHT must be laid down in an institutional protocol. Strong Agreement.

10) IHT should be taken into account and coded in the same way as other elements of clinical activity. Strong Agreement.

## Competing interests

The authors declare that they have no competing interests.

## Authors' contributions

JPQ, CM, AC, GC, OM, OF, and PYG contributed to the conception and design of the study, the draft and critical revision of the manuscript, and approved the final version.

## Authors' information

JPQ, CM, AC, GC, OM, OF and PYG for the expert panel: Dr. Claude Gervais (Nîmes), Pr. Jean-Christophe M. Richard (Rouen), Me Christelle Ledroit (Angers), Dr. Lionel Nace (Nancy), Dr. Julien Naud (Bordeaux), Mr. Stéphane Legoff (Paris), Mr. Kamel Touabi (Paris), Dr. Alexandre Ouattara (Bordeaux), Pr. Thomas Geeraerts (Toulouse), Dr. François Templier (Garches), Dr. Karim Tazarourte (Melun), Pr. Eric Roupie (Caen), Dr. Agnès Ricard-Hibon (Paris), Dr. Céline Farges (Paris).

JPQ, CM and AC are Presidents of the Organizing Committee and part of the Organizing committee for the "Commission des Référentiels et de l'Evaluation de la SRLF": M. Monchi, C. Bretonnière, K. Chaoui, A. Cravoisy, D. Da Silva, M. Djibré, F. Fieux, D. Hurel, V. Lemiale, O. Lesieur, M. Lesny, P. Meyer, C. Milesi, B. Misset, D. Orlikowski, D. Osman, J.P. Quenot, L. Soufir, T. Van der Linden, I. Verheyde.

GC, OM, OF and PYG are Expert Coordinators.

